# Supplementary figures and images for: Aortic Agatston score correlates with the progression of acute type A aortic dissection
Source: PLoS One. 2022 Feb 11;17(2):e0263881. doi: 10.1371/journal.pone.0263881 (PMC8836313; doi:10.1371/journal.pone.0263881)

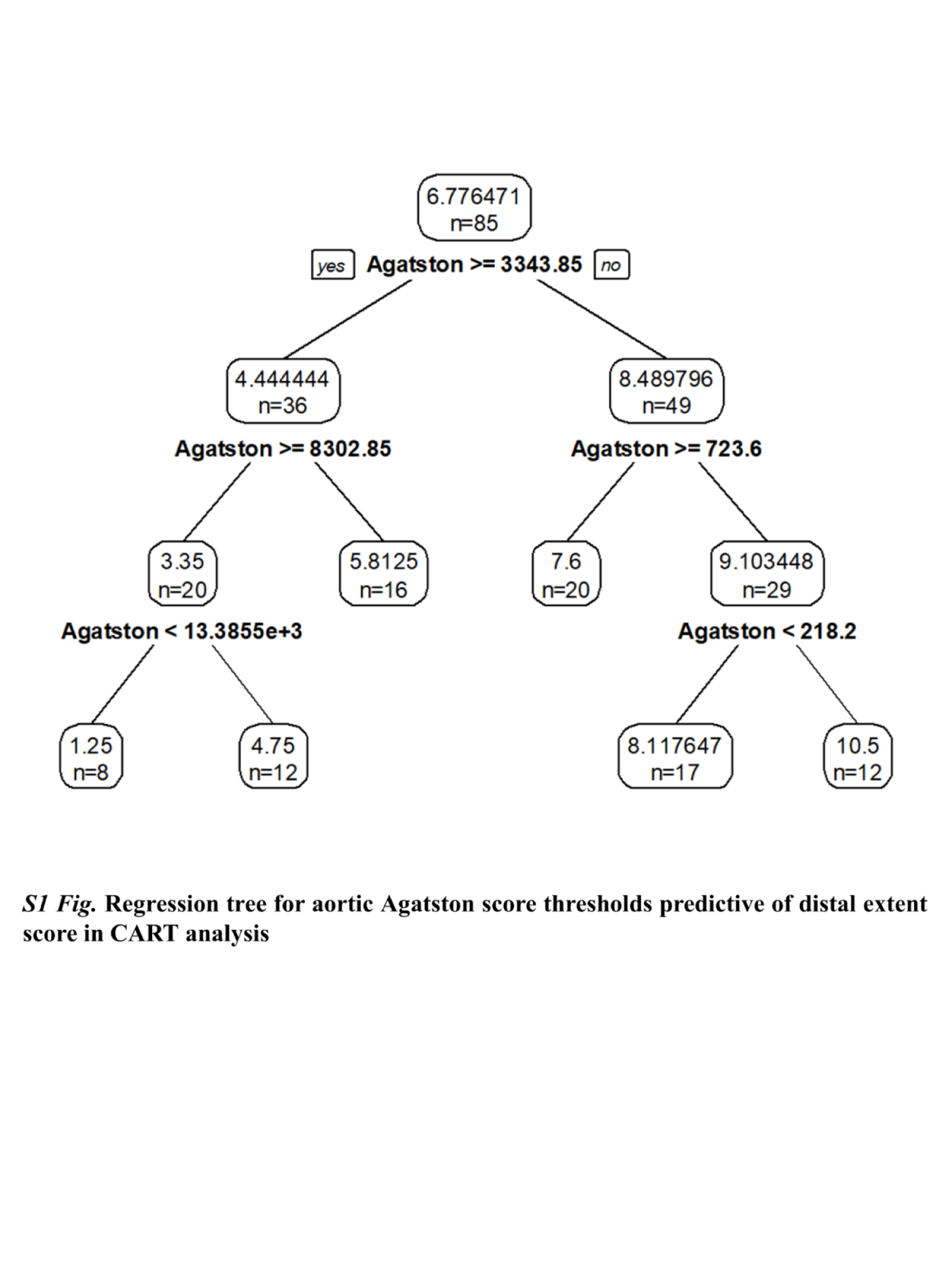

Supplement: S1 Fig — (TIFF) [file pone.0263881.s001.tiff]
