# Supplementary material for: Aortic Agatston score correlates with the progression of acute type A aortic dissection
Source: PLoS One. 2022 Feb 11;17(2):e0263881. doi: 10.1371/journal.pone.0263881 (PMC8836313; doi:10.1371/journal.pone.0263881)
Supplement: S1 Table — (DOCX) [file pone.0263881.s002.docx]

| **S1 Table. CTA variables of descending aorta before and after surgery in the patients with DeBakey Ⅰ or Ⅲb retrograde** | | | | | | |
| --- | --- | --- | --- | --- | --- | --- |
| Des CTA variables | Low-score group (n=40) | | *p*-value | High-score group (n=21) | | *p*-value |
|  | before surgery | after surgery |  | before surgery | after surgery |  |
| Distal extent score | 10 (8-11) | 10 (9–11) | 0.496 | 8 (5-9) | 8 (6–10) | 0.198 |
| Diameter (mm) | 30.9 (29.7-33.6) | 32.3 (31.1–34.4) | 0.077 | 30.7 (29.9-34.3) | 33.2 (30.6–36.3) | 0.199 |
| Area (mm^2^) | 796 (681-888) | 819 (760–895) | 0.136 | 763 (698-919) | 859 (715–1077) | 0.352 |
| True lumen area (mm^2^) | 256 (217-351) | 315 (240–482) | 0.058 | 385 (335-430) | 486 (377–663) | 0.038 |
| False lumen area (mm^2^) | 498 (426-604) | 496 (394–582) | 0.473 | 413 (337-533) | 327 (276–551) | 0.232 |
| True lumen/total lumen area ratio | 0.32 (0.28-0.4) | 0.4 (0.29–0.53) | 0.09 | 0.47 (0.38-0.58) | 0.6 (0.47–0.67) | 0.068 |

CTA, computed tomography angiography; Des, descending aorta.
