# Supplementary material for: Aortic Agatston score correlates with the progression of acute type A aortic dissection
Source: PLoS One. 2022 Feb 11;17(2):e0263881. doi: 10.1371/journal.pone.0263881 (PMC8836313; doi:10.1371/journal.pone.0263881)
Supplement: S2 Table — (DOCX) [file pone.0263881.s003.docx]

| **S2 Table. Early Postoperative Changes of CTA variables in the patients with DeBakey Ⅰ or Ⅲb retrograde (before - after surgery)** | | | | |
| --- | --- | --- | --- | --- |
| Postoperative Des CTA changes from before to after surgery | Total (n=61) | Low-score group (n=40) | High-score group (n=21) | *p*-value |
| Distal extent score difference  (before – after surgery) | 0 (-1-0) | 0 (-4-4) | -1 (-5-4) | 0.353 |
| Diameter fold-change  (after/before surgery) | 1.04 (1-1.08) | 1.04 (1-1.08) | 1.04 (1-1.06) | 0.873 |
| Area fold-change  (after/before surgery) | 1.04 (0.99-1.16) | 1.04 (0.99-1.16) | 1.03 (0.99-1.15) | 0.927 |
| True lumen area fold-change  (after/before surgery) | 1.23 (0.99-1.53) | 1.17 (0.96-1.77) | 1.27 (1.13-1.49) | 0.606 |
| False lumen area fold-change  (after/before surgery) | 0.93 (0.66-1.24) | 0.96 (0.77-1.18) | 0.76 (0.52-1.24) | 0.118 |
| True lumen/total lumen area ratio fold-change (after/before surgery) | 1.18 (0.87-1.57) | 1.14 (0.84-1.62) | 1.22 (1.01-1.48) | 0.649 |

CTA, computed tomography angiography; Des, descending aorta.
