# Supplementary material for: Aortic Agatston score correlates with the progression of acute type A aortic dissection
Source: PLoS One. 2022 Feb 11;17(2):e0263881. doi: 10.1371/journal.pone.0263881 (PMC8836313; doi:10.1371/journal.pone.0263881)
Supplement: S3 Table — (DOCX) [file pone.0263881.s004.docx]

| **S3 Table. Mid-term Postoperative Changes of plain CTA variables in the patients with DeBakey Ⅰ or Ⅲb retrograde in 6 months after surgery** | | | | |
| --- | --- | --- | --- | --- |
| Postoperative Des CTA changes in 6 months after surgery | Total (n=61) | Low-score group (n=40) | High-score group (n=21) | *p*-value |
| Diameter (mm^2^) | 32.4 (29.6-35.3) | 33.1 (30.2-36.9) | 31.7 (29.5-33.5) | 0.098 |
| Area (mm^2^) | 883 (766-1080) | 952 (784-1130) | 868 (753-997) | 0.182 |
| Diameter fold-change  / early postoperative diameter | 1 (0.93-1.07) | 1.01 (0.94-1.1) | 0.98 (0.87-1.04) | 0.058 |
| Area fold-change  / early postoperative area | 1.07 (0.97-1.25) | 1.11 (0.97-1.29) | 1.03 (0.88-1.13) | 0.031 |

CTA, computed tomography angiography; Des, descending aorta.
