# Supplementary material for: Aortic Agatston score correlates with the progression of acute type A aortic dissection
Source: PLoS One. 2022 Feb 11;17(2):e0263881. doi: 10.1371/journal.pone.0263881 (PMC8836313; doi:10.1371/journal.pone.0263881)
Supplement: Attachment — Submitted filename: PONE-D-21-14950_reviewer.docx [file pone.0263881.s005.docx]

The authors provide an interesting and potential important manuscript describing "Aortic Agatston score correlates with the progression of acute type A aortic dissection", The main issues concerning this paper are those concerning the potential associations between Aortic Agatston score and type A aortic dissection.

There are some weak points that need to be addressed by the authors

Major

1. Why Agatston Score 3344 is selected as the thresholds between the high and low scores of ATAAD.

2. Some recent literature on arterial dissection and calcification needs to be cited
